# Microtubules Are Essential for Mitochondrial Dynamics–Fission, Fusion, and Motility–in *Dictyostelium discoideum*

**DOI:** 10.3389/fcell.2016.00019

**Published:** 2016-03-22

**Authors:** Laken C. Woods, Gregory W. Berbusse, Kari Naylor

**Affiliations:** ^1^Department of Biology, University of Central ArkansasConway, AR, USA; ^2^Interdisciplinary Biomedical Sciences, University of Arkansas for Medical SciencesLittle Rock, AR, USA

**Keywords:** mitochondria, fission, fusion, cytoskeleton, latrunculin, nocodazole, *Dictyostelium discoideum*, CluA

## Abstract

Mitochondrial function is dependent upon mitochondrial structure which is in turn dependent upon mitochondrial dynamics, including fission, fusion, and motility. Here we examined the relationship between mitochondrial dynamics and the cytoskeleton in *Dictyostelium discoideum*. Using time-lapse analysis, we quantified mitochondrial fission, fusion, and motility in the presence of cytoskeleton disrupting pharmaceuticals and the absence of the potential mitochondria-cytoskeleton linker protein, CluA. Our results indicate that microtubules are essential for mitochondrial movement, as well as fission and fusion; actin plays a less significant role, perhaps selecting the mitochondria for transport. We also suggest that CluA is not a linker protein but plays an unidentified role in mitochondrial fission and fusion. The significance of our work is to gain further insight into the role the cytoskeleton plays in mitochondrial dynamics and function. By better understanding these processes we can better appreciate the underlying mitochondrial contributions to many neurological disorders characterized by altered mitochondrial dynamics, structure, and/or function.

## Introduction

Mitochondria are highly specialized eukaryotic organelles responsible for producing the majority of a cell's adenosine triphosphate (ATP). They also play a vital role in many other cellular processes, such as the synthesis of heme groups and the regulation of membrane potential, calcium homeostasis, apoptosis, and cell differentiation (Mitchell, 1961; Frezza et al., 2006; Lill and Mühlenhoff, 2008; Baughman et al., 2011; De Stefani et al., 2011; Martinou and Youle, 2011; Maeda and Chida, 2013). To carry out these cellular processes the mitochondria must function properly, which is largely controlled by the organelle's morphology and distribution throughout the cell (Nunnari and Suomalainen, 2012).

For instance, in several organisms like animals, flies, and yeast, as well as specialized neuronal cells, the mitochondria exist in a reticular dynamic network, while in organisms like *Dictyostelium* and *Arabidopsis* the mitochondria exist as individual organelles (Chen, 1988; Bereiter-Hahn, 1990; Nunnari et al., 1997; Rizzuto et al., 1998; Schimmel et al., 2012; El Zawily et al., 2014). In either case, the appropriate morphologies are maintained by fission and fusion events (Nunnari et al., 1997; Bleazard et al., 1999; Gilson et al., 2003; Karbowski and Youle, 2003; Twig and Shirihai, 2011; El Zawily et al., 2014).

The cytoskeleton, in addition to affecting mitochondrial morphology plays a crucial role in maintaining mitochondrial distribution throughout the cell by facilitating organelle transport to areas with high metabolic demands (Van Gestel et al., 2002; Bereiter-Hahn et al., 2008; Kostal and Arriaga, 2011; Nekrasova et al., 2011; Wu et al., 2013). In order to rapidly respond to cellular demands, the cytoskeleton must have a communication system allowing it to influence organelle transport and position. This system usually encompasses various linker and motor proteins. The interaction between the cytoskeleton and the mitochondria is poorly understood; however several studies suggest that cytoskeletal network proteins interact with components of the fission and fusion machinery (Liesa et al., 2009), as well with the calcium sensing GTPase- Miro (Fransson et al., 2006; Frederick and Shaw, 2007).

Mitochondrial dynamics (fission, fusion, motility) also regulate the organelle's morphology and distribution. Disruption of these dynamics has been linked to a loss of metabolic function, an increase in ROS concentration, impairment of ATP synthesis, and a decrease in overall membrane potential (Margolin, 2000; Karbowski and Youle, 2003; Baloh et al., 2007; Chen and Chan, 2009; Nunnari and Suomalainen, 2012; Youle and van der Bliek, 2012; Picard et al., 2013). A variety of diseases, such as Alzheimer's, Parkinson's, Charcot-Marie-Tooth 2A, and Huntington's have also been linked to disruption in mitochondrial dynamics and morphology, though the mechanism driving these afflictions are unknown (Nunnari and Suomalainen, 2012).

Thus, to better understand mitochondrial dynamics and their role in disease we utilized *D. discoideum*, a lower eukaryotic model organism. In addition to lower eukaryotes sometimes being easier to tease out molecular mechanisms, *D. discoideum* is also a mitochondrial disease system (Barth et al., 2007; Annesley and Fisher, 2009; Annesley et al., 2014). Mitochondrial diseases caused by a specific mutation often manifest with a variety of clinical symptoms in humans. It has become apparent that unlike humans, *D. discoideum* cells do not exhibit this variation in symptoms, thus simplifying the study on mitochondrial diseases (Francione et al., 2011).

In *D. discoideum* mitochondrial distribution is maintained by the protein CluA (Zhu et al., 1997), which has homologs across a variety of organisms, including *Arabidopsis thaliana, Saccharomyces cerevisiae*, and *Drosophila melanogaster*. In all organisms studied to date, the absence of this protein results in clustered mitochondria (Zhu et al., 1997; Fields et al., 1998; Logan et al., 2003; Cox and Spradling, 2009). In plants, the organelles are found in small distinct clusters distributed throughout the cell (El Zawily et al., 2014), while larger, nuclearly centered clusters have been identified in *D. discoideum*. Further work has shown that these *D. discoideum* mitochondria are interconnected by thin membranous strands with limited movement (Fields et al., 2002). It has been hypothesized that CluA may represent a novel family of proteins which link the cytoskeleton to mitochondria. In addition to clustered mitochondria, the absence of CluA also decreases the rates of fission and fusion (Schimmel et al., 2012). Further, the nuclear localization of the mitochondrial clusters suggest they only move in an anterograde fashion (Zhu et al., 1997). Interestingly the *D. discoideum* Miro homolog, GemA, is not involved in mitochondrial transport along the cytoskeleton (Vlahou et al., 2011); further supporting the notion that CluA links the cytoskeleton and mitochondria. Therefore, it is conceivable that CluA has direct influence on mitochondrial dynamics by association with the cytoskeleton; however, it is presently unclear if CluA interacts with actin or microtubule motor proteins. Thus, to determine CluA's function the relationship between mitochondria and the cytoskeleton in *D. discoideum* must be elucidated.

It has been demonstrated that in animal cells mitochondria move along microtubules to travel long distances and use actin filaments for short distances (Wu et al., 2013). *Drosophila* mitochondria primarily use microtubules (Cox and Spradling, 2009), as do fission yeast (Yaffe et al., 2003), whereas actin is predominantly used by both plants and budding yeast (Van Gestel et al., 2002). There is even evidence that mitochondria use intermediate filaments in 3T3 fibroblast cells for proper motility regulation (Nekrasova et al., 2011). Thus, we analyzed *D. discoideum* mitochondria to establish whether microtubules or actin filaments were utilized for proper motility. Additionally, we examined whether or not *cluA*^−^ mitochondrial morphology is dependent upon these cytoskeletal components and how these components affect motility of the *cluA*^−^ mitochondria. Finally, due to mounting evidence linking impaired fission and/or fusion to aberrant mitochondrial motility and cellular health (Cagalinec et al., 2013), we assessed the influence of the cytoskeleton on mitochondrial fission and fusion.

To carry out these experiments, we disrupted the microtubules with nocodazole and the actin filaments with latrunculin-B. Through immunofluorescence and time-lapse imaging, we quantified mitochondrial morphology, motility, and fission and fusion rates. Taken together our results show that the *cluA*^−^ clustered mitochondrial phenotype is partially dependent upon the actin and microtubule cytoskeletons, and that mitochondrial motility is not affected by loss of CluA. Therefore, we conclude that CluA does not play a significant role in connecting the mitochondria to the cytoskeleton. Further, we show that in *D. discoideum*, microtubules, but not actin, are important for both mitochondrial velocity as well as fission and fusion. Thus, we can infer that, as is the case with mammalian cells, microtubules play a much larger role in mitochondrial morphology and motility than actin. Finally, despite previous research, we did not find an interaction linking the mitochondria to the microtubule cytoskeleton through CluA, and conclude it may serve an unidentified function affecting mitochondrial morphology and distribution.

## Methods

### Strain culture and growth conditions

All *Dictyostelium discoideum* strains described were obtained from the Dicty-Stock Center (Fey et al., 2013). Wild-type (AX4) was deposited by Bill Loomis and *cluA*^−^ by Margaret Clarke. The strains were cultured axenically in liquid HL5 medium supplemented with streptomycin (final concentration of 300 ug/ml) and ampicillin (final concentration 150 ug/ml) at 22°C shaking at 125 rpm.

### Preparation of *D. discoideum* for experiments

AX4 and *cluA*^−^ cells were diluted to 3 × 10^4^ cells/ml in HL-5 liquid media until cells reached log phase. Log phase cells (5.0 ml) were washed by centrifuging at 500 × g for 4 min and resuspended in 5 ml of room temperature Lo-Flo (Formedium). Cells were stained with 0.1 uM MitoTracker CMXRos (Invitrogen) and incubated for 4 h at room temperature while shaking. Excess MitoTracker was removed by washing the cells twice with Lo-Flo.

### Cytoskeleton disruption

During the 4 h Lo-Flo incubation period, the drugs or their appropriate vehicle control were added to the cells to disrupt the cytoskeleton. To inhibit the actin portion of the cytoskeleton, 10 uM latrunculin-B (Sigma) or equivalent volume of vehicle control (EtOH) was added to the cells for the final 30 min of incubation in Lo-Flo media. Nocodazole (10 ug/ml) (Sigma) or a vehicle control of dimethyl sulfoxide (DMSO) (Sigma) was used to inhibit the microtubule component of the cytoskeleton and was added in the final hour of the 4-h incubation period. To inhibit both actin and microtubules, latrunculin-B (10 uM) and nocodazole (10 ug/ml) were used with the equal volume of ethanol and DMSO for a control. For washout experiments, after drug treatment cells were washed and then incubated at room temperature with shaking for 1 h prior to processing for immunofluorescence.

Following incubation, the cells were washed twice at 500 × g for 4 min to remove excess MitoTracker and resuspended in 5 ml Lo-Flo plus the appropriate drug or vehicle in preparation for live cell imaging or immunofluorescence. Drug effectiveness was confirmed with immunofluorescence (see below), in all drug treatments the cytoskeleton was significantly different from vehicle controls.

### Immunofluorescence of *Dictyostelium* strains

AX4 (wild-type) and *cluA*^−^ strains of *D. discoideum* were grown to log phase (about 2–4 × 10^6^) then pelleted at 500 × g for 4 min and resuspended in Lo-Flo liquid medium (Formedium). Cells were treated with MitoTracker, nocodazole, latrunculin-B, or both or treated with DMSO, ethanol, or both, as previously described, to disrupt the cytoskeleton and stain the mitochondria. Stained and treated cells were washed by pelleting at 500 × g for 4 min two times, and resuspended in room temperature Lo-Flo liquid media to the original volume. Drugs or control were added back to the washed and stained cells. A 22 × 22 mm coverslip was placed into a 6-well plate. About 500 ul of washed and stained cells were added to the coverslips and allowed to adhere for 30 min. The coverslips with adhered cells were then washed twice with 10 mM MES-NaOH by gently adding and removing the solution to remove any cells that did not adhere to the coverslips. The adhered cells were fixed with 1 ml of 3% paraformaldehyde diluted in 10 mM Pipes (pH 6.0) for 30 min and then quenched with 100 mM glycine (1 ml) diluted in 1xPBS for 5 min. The membranes of the adhered and fixed cells were then permeabilized by using 0.02% Triton X-100 for 5 min. The permeabilized cells were washed three times by gently adding and removing 1xPBS and then blocked with 0.045% fish gelatin, 0.5% BSA in 1xPBS (PBG) for 1 h at room temperature. These cells were prepared for either actin or tubulin visualization.

To visualize actin, the blocked cells were stained for 1 h at room temperature in the dark using 0.5 ul of 6.6 uM phalloidin (Life Tech) in 500 ul PBG per coverslip. Excess phalloidin was removed by washing with 1xPBS three times for 5 min each before mounting the coverslips with SlowFade Gold (Invitrogen) onto glass slides.

To visualize tubulin, tubulin primary antibodies (mouse anti-tubulin, DSHB 12G10) were diluted 1:150 in PBG and added to the coverslips. The primary antibodies were allowed to sit overnight at 4°C. The following day, the cells were washed with 1xPBS three times for 5 min each. The secondary antibody (AlexaFluor 488 goat α mouse IgG) (Life Tech A11001) diluted 1:250 in PBG was added and incubated in the dark for 1 h at room temperature. The coverslips with treated cells were then washed with 1xPBS three times for 5 min each and mounted to glass slides with SlowFade Gold.

### Quantification of morphology

**Figure 1:**
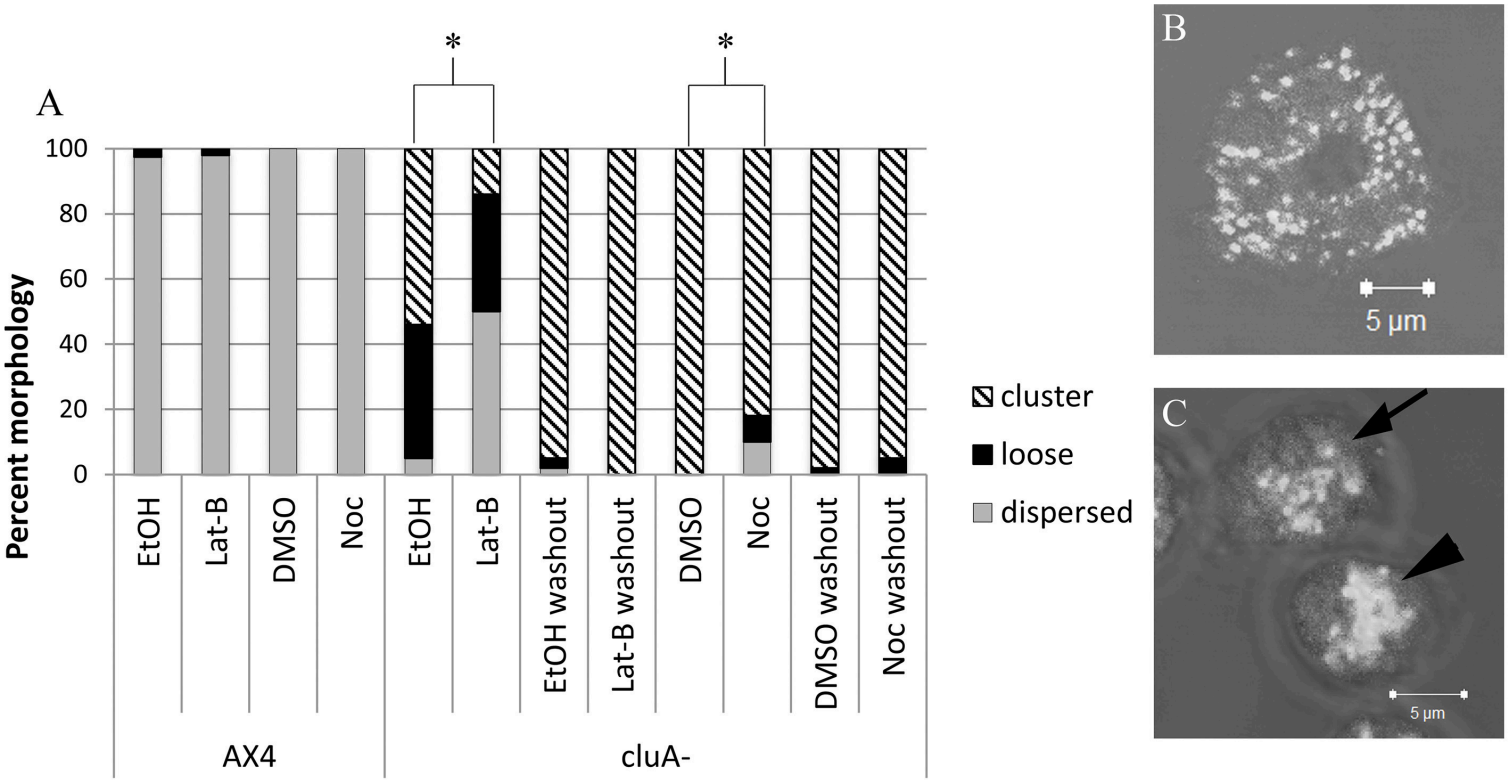
**Analysis of mitochondria distribution in AX4 and ***cluA***^−^ cytoskeleton disrupted cells. (A)** AX4 mitochondrial morphology remained dispersed and was unaffected by all treatments. The *cluA*^−^ clustered mitochondria phenotype was significantly decreased with more loose clusters and dispersed mitochondrial when treated with latrunculin-B (*p* < 0.0001) or nocodazole (*p* < 0.0001). **(B,C)** Examples of mitochondrial distribution. **(B)** AX4 cell with dispersed mitochondria, **(C)** *cluA*^−^ cells where top cell (with arrow) shows loosely clustered mitochondria, bottom cell (with arrowhead) shows a tight cluster. ^*^Indicates significant differences.

Cells were imaged using a Zeiss laser scanning LSM 510 Pascal confocal microscope to obtain z-stack images. The images were observed and classified according to the appearance of their microtubule and actin cytoskeleton and mitochondrial distribution. The microtubule cytoskeleton is present throughout the cell, while the actin localized around the cell at the membrane. The microtubule cytoskeleton morphology was classified as either little to none, patchy, or complete. Morphological classification was assessed by whether the microtubules appeared as disjointed and not extending throughout the cell (little to none), were in the astral configuration characteristic of the microtubule origin center (patchy), or branched throughout the cell and extending to the cell membrane (complete). The actin cytoskeleton was assessed by whether actin around the periphery of the cell was either absent or mostly absent (none), present but disjointed (patchy) or present and complete at the edge of the entire cell (complete). Mitochondrial morphology was determined to be distributed, loose cluster, or tight cluster (Figure 1). Distributed mitochondria were evenly dispersed throughout the cytoplasm, while clustered mitochondria were tightly aggregated toward the center or periphery of the cell. Loose clusters were considered loose mitochondrial aggregates.

### Statistical analysis-immunofluorescence

Each experiment was repeated a minimum of three times per condition with a minimum of forty cells being quantified. Statistical analysis was performed using Prism Graph Pad 6.07. A Chi square (or Fisher when appropriate) analysis was conducted to determine statistical significance among treatments and strains. A *p*-value of less than 0.05 was considered statistically significant.

### Quantification of mitochondrial fission and fusion in *Dictyostelium* strains

Washed and MitoTracker stained cells (0.5 ml) were placed in Nunc Lab-TekII 4-well chambered coverglass for imaging. A Zeiss laser scanning LSM Pascal confocal microscope with a pinhole setting of 144 um (1.36 airy units), resulting in an optical slice of 1.1 um was used to image washed, stained, and treated cells. A single plane was imaged every 677.38 ms for 100 time points, or until bleaching occurred.

To quantify fission events, mitochondria must be visible prior to the single organelle splitting into two. Fusion was quantified when two mitochondria approached and moved together for a couple of frames and then fused into a single organelle. If two organelles came together or split, then returned to their original state by the next frame, they were classified as “drive-bys” and were not quantified.

### Statistical analysis-fission and fusion

Statistical analysis was performed using JMP 11.0.0 (SAS Institute, Inc.) software. The rates of fission and fusion were calculated by averaging the number of events/min/cells for each strain and treatment. A minimum of 30 cells for each strain was used for quantification. Kruskal-Wallis analysis with a Steel-Dwass *post-hoc* was performed for statistical analysis. A *p*-value of less than 0.05 was considered statistically significant.

### Kymograph generation and motility analysis

Kymographs were generated using ImageJ from single plane confocal time lapse images (Schneider et al., 2012). A region of interest (ROI) was selected within a cell in the first image of each series. ROIs were drawn through the left, middle, and right portions of the cell in every instance where cells were visible throughout the entire series of images. The ROIs were stacked and converted to generate kymographs that depict mitochondrial movement within the region of interest over time. Kymographs were generated for a minimum of 20 cells in each treatment for AX4 and *cluA*^−^ strains. To quantify motility, the speed in pixels/0.677 s was calculated and converted to micrometers/second for comparisons. Mitochondrial motility from the left, middle, and right portions were calculated and averaged for each cell. Kruskal-Wallis with Steel Dwass *post-hoc* analysis was conducted using JMP software for statistical comparisons of single drug vs. single control. A *p*-value of less than 0.05 was considered statistically significant.

The percent of mitochondria moving were counted from the kymographs created for motility analysis. Mitochondria in each kymograph were counted and classified as either moving or not moving, with stationary mitochondria being considered a straight vertical line from the top of the kymograph to the bottom. An average percent of mitochondria moving was calculated for each strain and treatment and compared; a minimum of 20 cells were analyzed for each treatment. For analysis, nonparametric Kruskal-Wallis and *post-hoc* Steel-Dwass statistical tests were used. A *p*-value of less than 0.05 was considered statistically significant.

## Results

### The relationship between the cytoskeleton and mitochondrial morphology in *D. discoideum*

We quantified mitochondrial morphology after disrupting actin with latrunculin-B (Lat-B) or microtubules with nocodazole (Noc) in wild-type (AX4) and *cluA*^−^ strains. The morphology and motility rates were compared to respective controls, as well as comparisons within treatment and across strains. These comparisons allowed us to determine the effect of each component of the cytoskeleton on mitochondrial morphology in *cluA*^−^ cells.

As expected, alteration of the cytoskeleton with these pharmaceuticals does not affect mitochondrial distribution in AX4 cells when compared to vehicle control cells (Noc: *p* = 1.0; Lat-B: *p* = 0.9999; Figure 1). However, disruption of the cytoskeleton did alter mitochondrial distribution in *cluA*^−^ cells. Treatment with either nocodazole or latrunculin-B changed *cluA*^−^ mitochondria from their characteristic clustered morphology to a higher prevalence of loose clusters (Noc: *p* < 0.0001; Lat-B: *p* < 0.0001; Figure 1). As a further control, we treated the cells with vehicle or drugs then washed the cells and quantified mitochondrial morphology. Results from washout experiments indicate that indeed all changes to mitochondrial morphology are specific to the treatments, though interestingly the vehicles themselves have some effect (Figure 1). Especially EtOH, which increases the number of loose mitochondria compared to the washout controls. To determine if there was a synergistic effect between actin and microtubules, mitochondrial morphology of both strains was analyzed when the cells were exposed to nocodazole and latrunculin-B simultaneously. Our results indicate that there is no significant synergistic effect (data not shown), thus we can conclude that both cytoskeletal filaments are involved in maintaining the tightly clustered *cluA*^−^ phenotype but do not affect the wild-type dispersed mitochondrial phenotype.

### The relationship between the cytoskeleton and mitochondrial motility in *D. discoideum*

To determine the role of actin and microtubules in mitochondrial motility, cells were treated with cytoskeletal disrupting drugs and mitochondrial velocity and the percentage of organelles moving was calculated from kymographs. Again we analyzed both AX4 wild-type cells and *cluA*^−^ cells, to determine not only the role of the cytoskeleton but also the role CluA may play in mitochondrial motility.

**Figure 2:**
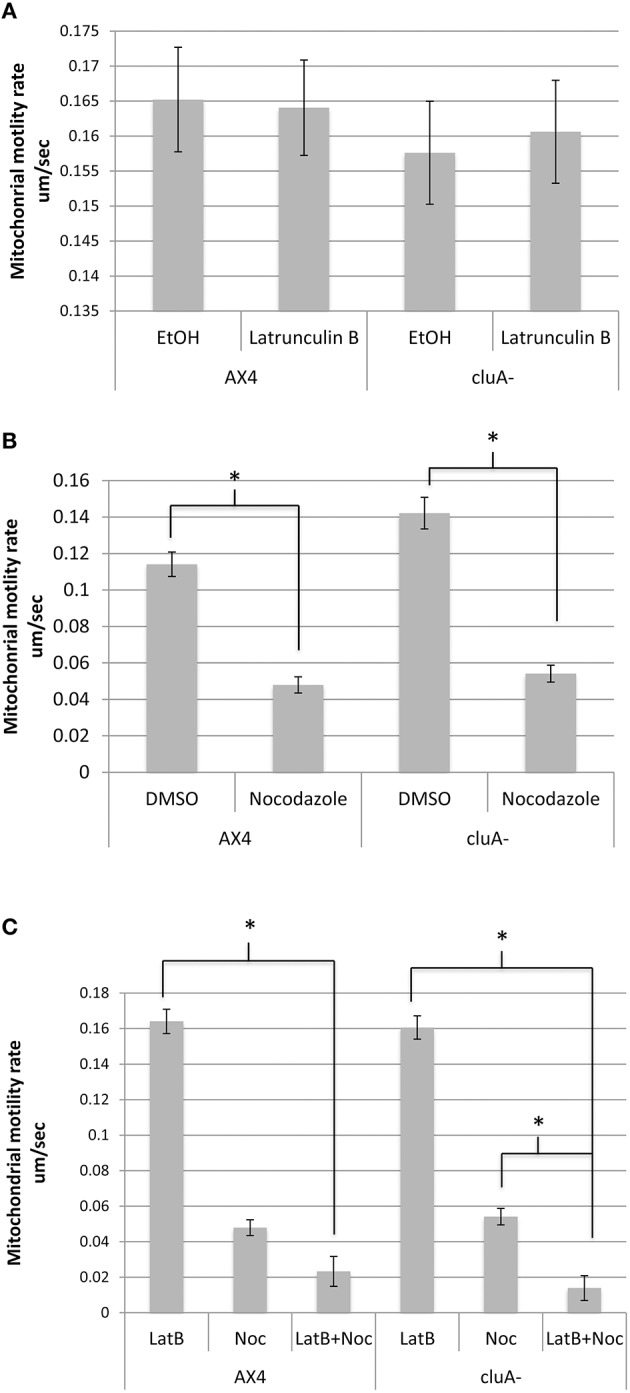
**Average mitochondrial speed compared across strains and treatments. (A)** There was no significant difference between treatments in AX4 (*p* = 1.0) or *cluA*^−^ (*p* = 1.0) when treated with latrunculin-B. Comparing the treatments across strains also failed to prove statistical significance in ethanol (*p* = 1.0) and latrunculin-B (*p* = 1.0) treatments. **(B)** AX4 and *cluA*^−^ mitochondria treated with nocodazole moved significantly slower than in vehicle treated cells (AX4, *p* < 0.0001; *cluA*^−^*, p* < 0.0001). AX4 DMSO mitochondrial speed was not significantly slower than the *cluA*^−^ DMSO treatment (*p* = 0.57) or nocodazole treatment (*p* = 0.99). **(C)** Cells were treated with nocodazole and latrunculin-B simultaneously to determine if there was a synergistic effect between actin and microtubules. In AX4 only latrunculin-B (*p* < 0.0001) but not nocodazole (*p* = 0.29) single treatments had significantly higher mitochondrial rates compared to the double treatment. In *cluA*^−^ both single treatments (Lat-B: *p* < 0.0001; Noc: *p* = 0.0031) had significantly higher motility rates compared to the double drug treated *cluA*^−^ cells. CluA plays no direct role in mitochondrial motility, while microtubules determine the speed of mitochondrial movement; though in the absence of CluA, actin does play a significant role also. ^*^Indicates significant differences.

When actin is inhibited with latrunculin-B, AX4 had an average mitochondrial speed of 0.164 ± 0.007 um/s while the ethanol control averaged a speed of 0.165 ± 0.007 um/s, with no statistical difference between treatments (*p* = 1.0; Figure 2A). Similarly, the *cluA*^−^ latrunculin-B and control treated cells averaged mitochondrial speeds of 0.161 ± 0.007 um/s and 0.158 ± 0.007 um/s, respectively, with no statistical significance (*p* = 1.0; Figure 2A). A comparison of treatments across strains also proved to not be statistically significant for latrunculin-B (*p* = 1.0) and ethanol (*p* = 1.0; Figure 2A). Thus, neither actin nor CluA has a significant role in mitochondrial velocity.

When measuring motility for microtubule inhibited cells, there was a significant difference between the drug treated cells and their control for both strains. In AX4, the DMSO control had an average rate of 0.1141 ± 0.007 um/s while the nocodazole treated mitochondria moved 55% slower at 0.0479 ± 0.004 um/s (*p* < 0.0001). Similarly, in *cluA*^−^, mitochondrial speed in the control was 0.1422 ± 0.009 um/s and 0.0541 ± 0.005 um/s in the nocodazole treated cells, a 64% reduction in mitochondrial speed than the control cells (*p* < 0.0001; Figure 2B). Comparing treatments across strains proved that there was no significant difference in nocodazole or DMSO treated AX4 and *cluA*^−^ (Noc: *p* = 0.99; DMSO: *p* = 0.57), though the DMSO vehicle AX4 cells had a 21% slower mitochondrial speed than DMSO *cluA*^−^ (Figure 2B).

Again we determined if there was a synergistic effect between microtubules and actin in terms of mitochondrial velocity. In AX4, there is no statistical difference between nocodazole treatment and double drug treatment (*p* = 0.29; Figure 2C). Interestingly, in *cluA*^−^ cells**, nocodazole treated cells had a statistically higher motility rate, by 72%, than the double drug treated cells (*p* = 0.0031; Figure 2C). These results indicate that microtubules have the largest role in velocity, but when actin, microtubules, and CluA are disrupted, it is apparent that actin also has a contributory effect.

**Figure 3:**
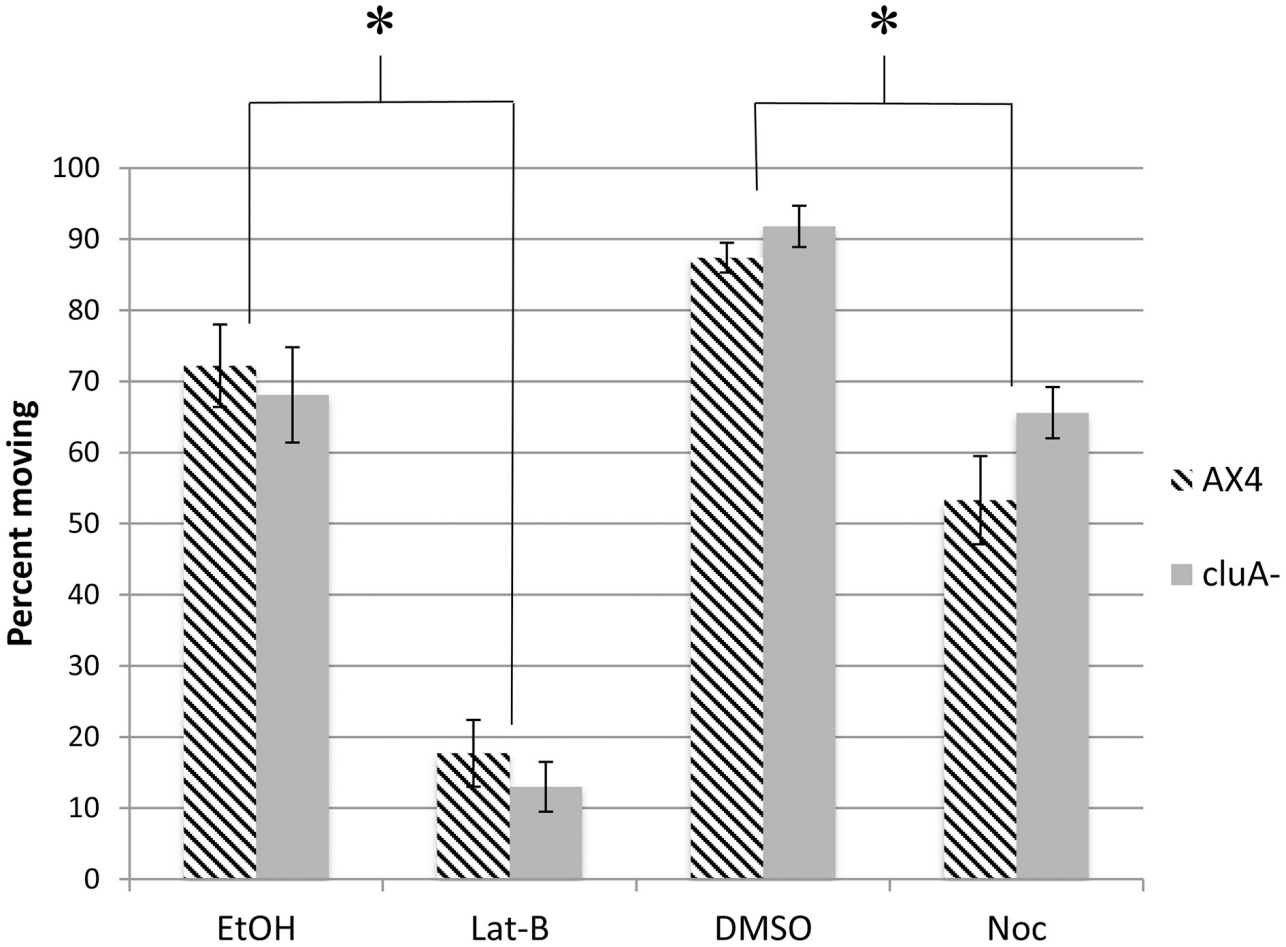
**Percent of mitochondria moving in cytoskeleton disrupted AX4 and ***cluA***^−^ strains**. In AX4 cells, all drug treatments significantly lowered the percent of mitochondria moving in comparison to their appropriate controls (Lat-B: *p* < 0.0001; Noc: *p* = 0.0005). This also occurred in the *cluA*^−^ strain (Lat-B: *p* < 0.0001; Noc: *p* < 0.0013). Inhibiting the cytoskeleton significantly decreased the percent of mitochondria moving in both wild-type and *cluA*^−^ strains. ^*^Indicates significant differences.

In addition to measuring the speed of mitochondrial movement we also quantified the percent of mitochondria moving from the kymographs. Approximately 72 and 87% of mitochondria are moving in the vehicle control EtOH and DMSO treated AX4 cells respectively (Figure 3). When treated with latrunculin-B, the percent of mitochondria moving was reduced by about 75% (*p* < 0.0001), while nocodazole treatment reduced the number of moving mitochondria by 39% (*p* < 0.0005). Further analysis showed that there is no synergism between microtubules and actin for determining how many mitochondria are moving. Thus, both actin and microtubules are necessary for determining how many mitochondria move, but actin is likely the predominant cytoskeletal element.

For *cluA*^−^ single vehicle controls, 92% of mitochondria were moving in DMSO treated cells, with 68% moving in ethanol. When microtubules were inhibited, the percent of mitochondria moving was reduced by 28% (*p* < 0.0013), while inhibiting actin reduced the number of moving mitochondria by 81% (*p* < 0.0001; Figure 3). Again there is no synergistic effect, thus these results suggest in *cluA*^−^**, as in AX4, that actin plays the predominant role in the percent of moving mitochondria but both cytoskeletal elements are involved.

Overall, both actin and microtubules are necessary for mitochondrial motility in *D. discoideum*, while CluA seems to have no significant role. Our results indicate that microtubules play the largest role in determining velocity of movement, while actin seems to be more important for how many mitochondria are moving.

### Assessing the relationship between the cytoskeleton and mitochondrial fission and fusion in *D. discoideum*

Little is known about the cytoskeletal influences on mitochondrial fission and fusion but it is expected that the cytoskeleton would play a critical role. To give more insight to this potential interaction, we quantified both fission and fusion rates in the wild-type and *cluA*^−^ strains when the microtubules or actin filaments were disrupted. The fission and fusion rates of drug treated cells were compared to an appropriate vehicle control, as well as across strains. Comparing these fission and fusion rates allowed us to determine the role of the cytoskeleton in *D. discoideum* fission and fusion.

Using laser scanning confocal microscopy, a series of single plane images of *D. discoideum* were captured that showed the real time mitochondrial movement in the cells. Fission and fusion events were quantified in each cell and a rate was calculated. When the actin cytoskeleton was inhibited in AX4 cells, fission (*p* = 1.0) and fusion (*p* = 1.0) rates were not significantly different compared to the control, additionally fission and fusion remained balanced within each treatment for both latrunculin-B (*p* = 1.0) and ethanol (*p* = 1.0) treated cells (Table 1).

**Table 1:** **Data table for the comparison of fission and fusion rates, with standard error, of all AX4 treatments**.

**Treatment**	**Fission rate (events/min/cell)**	**Fusion rate (events/min/cell)**
AX4 DMSO	0.79 ± 0.15	0.79 ± 0.14
AX4 Nocodazole	0.12 ± 0.04	0.15 ± 0.05
AX4 EtOH	0.89 ± 0.1	0.86 ± 0.1
AX4 Latrunculin-B	0.75 ± 0.1	0.74 ± 0.09

The AX4 microtubule cytoskeleton was inhibited using nocodazole. In these cells the rates of fission and fusion remained balanced (Noc: *p* = 1.0; DMSO: *p* = 1.0; Table 1) though, it was apparent that inhibiting microtubules significantly lowered fission by 85% (*p* = 0.004) and fusion by 81% (*p* = 0.003; Table 1). Further analysis demonstrated there was no synergistic effect between microtubules and actin in these processes (data not shown), thus microtubules are required for mitochondrial fission and fusion while actin plays little to no role in these processes.

As the cytoskeleton is required for fission and fusion, we wondered if disrupting the cytoskeleton would also decrease the rates of fission and fusion in cells lacking CluA. Our results show that DMSO treated *cluA*^−^ mitochondria fission and fusion rates were balanced (*p* = 1.0) and similar to the ethanol control rates, which were also balanced (*p* = 1.0; Table 2). *cluA*^−^ strains treated with nocodazole showed significantly lower fission and fusion compared to the DMSO control, 81 and 78% respectively (fission, *p* < 0.0001; fusion, *p* < 0.0001; Table 2). Within treatment, the rates of fission and fusion remained balanced (Noc: *p* = 1.0; Table 2). When treated with the actin inhibiting drug latrunculin-B, there was no difference in fission (*p* = 1.0) or fusion (*p* = 1.0) when compared to the ethanol control (Table 2). Again, as in wild-type cells, further analysis demonstrated there was no synergistic effect between microtubules and actin in fission and fusion in *cluA*^−^ cells (data not shown). Thus, the microtubules exert a greater influence on regulating mitochondrial fission and fusion. Moreover, actin filaments were found to have no significant effect, even in the absence of CluA.

**Table 2:** **Data table for the comparison of fission and fusion rates, with standard error, of all ***cluA***^−^ treatments**.

**Treatment**	**Fission rate (events/min/cell)**	**Fusion rate (events/min/cell)**
*cluA^−^* DMSO	0.97 ± 0.1	0.87 ± 0.1
*cluA^−^* Nocodazole	0.18 ± 0.08	0.19 ± 0.08
*cluA^−^* EtOH	0.81 ± 0.09	0.71 ± 0.08
*cluA^−^* Latrunculin-B	0.67 ± 0.09	0.57 ± 0.09

## Discussion

### The role of microtubules in mitochondrial dynamics

Here we present data suggesting that microtubules are the predominant cytoskeletal element for moving and distributing *D. discoideum* mitochondria. We show an almost complete loss of motility in cells treated with nocodazole as well as the relaxed and more distributed clusters found in *cluA*^−^ cells with disrupted microtubules. Additionally we and others have observed a small population of mitochondria that associate with the microtubules (Vlahou et al., 2011). Finally work by Vlahou et al demonstrates that mitochondrial distribution is dependent upon intact microtubules (Vlahou et al., 2011).

We also demonstrate that microtubules are essential for mitochondrial fission and fusion. It has not yet been teased out whether disruption of fission and fusion in these cells prevents motility or if motility must be functional for fission and fusion to take place. It has been suggested that blocks of fission and fusion will inhibit motility and distribution. Incomplete fission can result in a tangle of interconnected mitochondria and incomplete fusion can result in mitochondrial aggregates, thus motility's effect on the processes seems clear (Chen and Chan, 2009). On the other hand, it has been shown that loss of Miro, which inhibits motility, subsequently inhibits fusion (Cagalinec et al., 2013). It is logical to assume that motility facilitates fission and fusion as at least one mitochondrion must move toward another for fusion to take place and once divided the organelles must move apart to remain separate. Either way it is apparent that mitochondrial dynamics are intimately linked to motility and in *D. discoideum*, as suggested by our data, regulated by microtubules.

### The role of actin in mitochondrial dynamics

Our results suggest that disruption of actin decreases the number of mitochondria moving, but the ones that are moving, move at the same speed and go to the same locations as in untreated cells. This suggests to us that while actin may not be a major highway for mitochondrial movement it may function as an entrance ramp, helping mitochondria get to the highway as needed. If this is the case, it is apparent that by actively targeting mitochondria, the cell can select the organelles that need to be transported to the sites of high energy needs or perhaps undergo fission and fusion to repair mitochondrial DNA preventing a buildup of damaged or older mitochondria. Microtubules can then move the selected mitochondria and regulate fission and fusion events. This model is similar to mitochondrial behavior in neurons. Neurons utilize the actin cytoskeleton to move mitochondria shorter distances and microtubules for long distance transport (Morris and Hollenbeck, 1995). The shorter distance movement is due to the neuron's need to retain mitochondria at sites of high ATP utilization (Boldogh and Pon, 2006).

### The role of CluA in mitochondrial dynamics

CluA is required for distribution and plays a role in fission and fusion. Our results indicate that tight cluster formation is dependent upon CluA, microtubules, and actin, but CluA is not a significant player in mitochondrial motility. Therefore, CluA is most likely not an adaptor protein linking mitochondria to the cytoskeleton. Instead we suggest that *D. discoideum* must have a novel adaptor protein not yet identified; perhaps an intermediate filament as indicated in 3T3 fibroblast cells (Nekrasova et al., 2011).

Interestingly while we suggest CluA is not a linker protein, the cytoskeleton does appear to play a larger role in mitochondrial distribution and motility when CluA is absent. The clustered phenotype of *cluA*^−^ cells is relaxed by the disruption of the actin and microtubule cytoskeletons, and there is a synergistic decrease in motility when CluA, microtubules, and actin are all disrupted. Perhaps this is simply a result of mis-regulation of fission and fusion in *cluA*^−^ cells.

In conclusion, *D. discoideum* mitochondria move along the microtubule cytoskeleton, similar to what is reported in animal cells, and without this movement mitochondrial fission and fusion cannot take place. Finally, we propose that the link between mitochondria and the microtubules is not CluA and that this protein plays an as yet unidentified role in mitochondrial fission and fusion.

## Author contributions

LW contributed to the acquisition, analysis and interpretation of the data, she drafted, revised, and approves the final version of the manuscript. GB contributed to the design and acquisition of the work. He revised and approves the final version of the manuscript. KN contributed to the design, acquisition, and interpretation of the work. She drafted, revised, and approves the final version of the manuscript. LW, GB, KN agree to be accountable for all aspects of the work.

### Conflict of interest statement

The authors declare that the research was conducted in the absence of any commercial or financial relationships that could be construed as a potential conflict of interest.
